# Family Members’ Perspectives on Family and Social Support Available to Suicidal Patients, and Health Systems’ Interactions and Responses to Suicide Cases in Alberta: Protocol for a Quantitative Research Study

**DOI:** 10.2196/19112

**Published:** 2020-11-24

**Authors:** Rabab M Abou El-Magd, Liana Urichuk, Shireen Surood, Daniel Li, Andrew Greenshaw, Mara Grunau, Laureen MacNeil, Ione Challborn, David Grauwiler, Robert Olson, Vincent Israel Opoku Agyapong

**Affiliations:** 1 Department of Psychiatry Faculty of Medicine University of Alberta Edmonton, AB Canada; 2 Addiction & Mental Health, Alberta Health Services Edmonton, AB Canada; 3 Centre for Suicide Prevention Calgary, AB Canada; 4 Canadian Mental Health Association Calgary, AB Canada; 5 Canadian Mental Health Association Edmonton, AB Canada

**Keywords:** suicide in Alberta, suicide, family members’ perspectives, social support, health systems interactions

## Abstract

**Background:**

Suicide is a major cause of preventable death globally and a leading cause of death by injury in Canada. To support people who experience suicidal thoughts and behaviors and to ultimately prevent people from dying by suicide, it is important to understand individual and familial experiences with the health care system.

**Objective:**

We present the protocol for a study, the objective of which is to explore how people who died by suicide, and their family members, interacted with the health care system.

**Methods:**

This is a quantitative research study. Data will be collected through a self-administered paper-based or online survey of the family member of patients who died by suicide. The sample size was calculated to be 385 (margin of error ±3%).

**Results:**

Data collection will start in October 2020 and results will be available by March 2021. We expect the results to shed light on the experiences of individuals who died by suicide and their family members with the health care system. The study has received ethical clearance from the Health Ethics Research Board of the University of Alberta (Pro00096342).

**Conclusions:**

Our study may inform practice, policy, and future research. The findings may shape how members of the health care system respond to people who are at risk of suicide and their families.

**International Registered Report Identifier (IRRID):**

PRR1-10.2196/19112

## Introduction

### Background

Suicide is a serious global public health problem, with an estimated 800,000 people reported to die by suicide every year [1]. In Canada, suicide remains the 9th leading cause of death and the second leading cause of death among children, youth, and young adults [2]. Suicide impacts people of all ages and backgrounds in Canada. Every day, an average of more than 10 Canadians die by suicide. There are close to 6000 emergency department visits and 2000 hospitalizations every year for self-inflicted injuries [3]. For every person lost to suicide, many more experience thoughts of suicide or suicide attempts. For every death by suicide, a large circle of survivors are significantly affected by the loss. Each suicide results in 135 people exposed (ie, who knew the person), who may need clinician services or support following exposure [4].

There were 4000 suicides in Canada in 2018 [5], with more than 500 of these deaths occurring in Alberta. Suicide is consistently a leading cause of death among Albertans. Suicide claims more lives annually than other causes such as motor vehicle collisions and homicides. Over 75% of those deaths occur among men, most between the ages of 30-69 years [6]. Health care systems play a vital role in suicide prevention. One study in Alberta, for instance, found that the majority of people who died by suicide used a health service in the year prior to their death. They were also more likely to use the emergency department, in-patient services, or community mental health services than those who died from other causes; they typically used health services for mental disorders as well [7].

In Alberta, which is the site of this study, suicide prevention initiatives, including Living Hope, are underway to enhance aspects of the health care system, as evidenced by the Implementation Plan for the Edmonton Suicide Prevention Strategy [8]. Living Hope promotes a comprehensive preventative approach that seeks to enhance access to the protective factors that decrease the risk of suicide. The implementation plan upholds the inherent value of every person and recognizes that residents of Alberta, both as service providers and as community members, can offer the compassion, respect, and hope needed to increase resilience and nurture hope for those contemplating suicide [8].

The Mental Health Commission of Canada, in collaboration with the Canadian Association for Suicide Prevention, the Centre for Suicide Prevention, the Public Health Agency of Canada, alongside an Advisory Committee comprising people with lived experience related to suicide, have developed toolkits to support individuals who have been impacted by suicide. One toolkit is tailored for people who have attempted suicide, and the other is focused on resources for people who have lost someone to suicide [9].

Beyond stakeholder engagement [10] and an understanding of the dimensions of service quality [11], little is known locally about the personal, family, and social circumstances of people who died by suicide in Alberta. Similarly, little is known about how individuals who died by suicide and those close to them experienced the health care system. The current mechanism by which Alberta Health Services (AHS) investigates suicide is through the Quality Assurance Review (QAR). A QAR of an adverse event utilizes the Systems Analysis Methodology, which aims to determine what happened, how it happened, and what can be done to improve care for future patients. This type of review generally involves engaging a multidisciplinary team to examine all of the health care system components (eg, environment, task, policy, etc) as they relate to an event (or group of similar events). This process often results in recommendations aimed at improving the quality and safety of health care delivery. The focus is on improving structures, processes, and/or practices within AHS [12]. QARs are done following a suicide on a case-by-case basis, and the results are not shared beyond those directly involved. The privacy of the QAR limits case comparison and knowledge translation. Additionally, an understanding of the context of death by suicide is needed, as it is thought to differ from the context of a suicide attempt. QARs usually focus on the health systems’ contributions to the suicide and do not place much emphasis on examining the personal, familial, and societal factors that also contribute to deaths by suicide. One study found that while individuals who attempt suicide generally exhibited similar levels of depression, those who died by suicide were significantly more likely to have experienced significant job stress and financial problems, left a suicide note, and used alcohol and drugs prior to the act [13]. AHS is committed to patient- and family-centered care [14], which highlights the importance of talking to families about both their own and their relatives’ experiences with the health care system. Ultimately, insight into the experiences of people who died by suicide, and their family members, has the potential to inform policy and practice, and shape how members of the health care system, and AHS specifically, respond to individuals who are at risk of suicide.

### Objectives

The purpose of this study is to understand better the family and social circumstances of individuals who died by suicide, and how those who died by suicide and their family members interacted with the health care system. This study extends the knowledge to be gained from a recently completed qualitative study that examined family members’ perspectives on health system interactions with those who died by suicide [15].

Our specific quantitative research questions are:

1. What factors related to family, society, and health systems contribute to death by suicide in Alberta?
2. How do individuals impacted by the suicide of a family member perceive their own interactions with the health care system?

To the best of our knowledge, no previous province-wide study has examined the personal, familial, societal, and health systems factors that contribute to suicide deaths in Canada. One study was conducted by Schaffer et al [16] to investigate the population-based analysis of health care contacts among suicide decedents prior to death by suicide. It was a systematic extraction of data from records at the Office of the Chief Coroner of Ontario of each person who died by suicide in the city of Toronto from 1998 to 2011 [16]. Consequently, this work, the first of its kind in Alberta and in Canada, could help identify important factors that are associated with deaths by suicide in the province of Alberta.

## Methods

### Study Design

This study utilizes a quantitative research design. Data will be collected through a self-administered paper-based or online survey of the family members of patients who died by suicide (Multimedia Appendix 1). A sample size of 385 was predetermined on the assumption that with an annual average of 500 people dying by suicide in Alberta, a 95% CI, and one family member per suicide decedent completing the survey, the sample size needed to estimate family members’ perspectives on health system interactions, as well as family and social support for suicidal patients, with the margin of error ±3%, is 385. Data will be collected via both paper format and online. Prospective participants will be provided with paper-based or online information leaflets.

### Participants

Participants will be adults; they should also have a close family member who has died by suicide in the previous 12 months and had regular contact with this family member prior to their suicide, such that they are reasonably aware of their personal, family, and social situation prior to their suicide as well as their interaction with the health care system. Participants do not have to identify themselves and their submission of the survey implies their consent.

### Data Collection

We initially designed a survey form that reflected risk factors for dying by suicide identified in the published literature as well as additional factors to help answer our research questions. The draft survey questions were reviewed by the Canadian Mental Health Association (CMHA), Alberta Division, and the Centre for Suicide Prevention, and changes were made based on the feedback received. The survey was then pretested on two volunteer family members of patients who had died by suicide before being further revised and finalized for use in the study. The survey questions take 10-55 minutes to complete, and no incentives will be offered to participants who complete the survey. Paper-based recruitment will be done in collaboration with the CMHA regional offices in Alberta. The association runs focus groups for family members of people who died by suicide, with hundreds of people attending annually. Information leaflets and posters advertising the study will be distributed among prospective participants attending these focus groups. Those interested in participating in the study will be provided with guidance on how to access the survey questions.

In addition, online versions of the survey will be promoted through the websites and social media feeds of AHS, the CMHA, the Centre for Suicide Prevention, the Edmonton Mental Health Foundation, and the University of Alberta’s Faculty of Medicine and Dentistry. The online survey is designed in accordance with the CHERRIES (Checklist for Reporting Results of Internet E-Surveys) checklist [17]. Prospective participants will be invited to review the online version of the information leaflet and proceed to complete the survey. Information identifying participants will not be collected for the online survey, and completion and submission of the survey denotes consent. The study will be conducted in accordance with the Declaration of Helsinki (Hong Kong Amendment) and Good Clinical Practice (international guidelines). Informed consent will be obtained from each participant. The study has received ethical clearance from Health Ethics Research Board of the University of Alberta (Pro00096342).

### Data Analysis

Quantitative data will be analyzed using SPSS, version 26 (IBM Corp), using descriptive statistics and correlational analyses [18]. A chi-square test will be used to explore differences in responses between demographic variables of the suicide decedent and the respondent.

## Results

Data collection is expected to commence in October 2020. Results will be available by March 2021. Findings from the study will help illuminate factors related to family, society, and health systems, and the role they play in death by suicide in Alberta.

The study results will be disseminated at several levels, including to participants, practitioners, academics/researchers, and health care organizations.

Our team will plan an organizational engagement strategy to advance discussions about feasibility and effectiveness prior to the conclusion of the trial. This will help ensure the findings are a relevant part of decision-making processes. In addition, this may facilitate the planning of a larger study that is endorsed at both leadership and operational levels so that the potential benefits of the study results can reach participants in a timelier fashion.

## Discussion

The main objective of this study is to investigate the familial, societal and health systems–based support available to individuals who die by suicide in Alberta. It also aims to examine the support offered by the health care system in Alberta to family members of patients who die by suicide.

Studies in other jurisdictions suggests that personal, familial, and social factors such as stigma [1], public education [19,20], psychiatric illness [21,22], age [23,24], gender [1,23], marital status [25], positive support [26,27], familial history of suicide [28-31], and alcohol consumption [32,33] are associated with death by suicide. Similarly, health system factors such as staff attitude toward suicidal persons [19], recency of hospitalization for suicide attempt and recent health care contact [19,21,34-37], underdiagnoses of mental disorders and major depressions [38], brevity of interactions with medical staff [39], ignoring suicide-related warning signs by health care providers [40], lack of trust in health care services [41], and relatives’ feelings of exclusion from information on treatment [42] have all been positively associated with deaths by suicide in studies conducted in other jurisdictions. The results of our study will provide us with information on familial, societal, and health systems–related influences in Alberta as well as aspects of care in need of further improvement and refinement. The recommendations arising from this study have the potential to lead to significant system enhancements and reductions in suicide rates in Alberta and beyond.

## Appendix

Multimedia Appendix 1 Quantitative research study survey.

## Abbreviations

AHS: Alberta Health Services
CHERRIES: Checklist for Reporting Results of Internet E-Surveys
CMHA: Canadian Mental Health Association
QAR: Quality Assurance Review
