# Supplementary material for: Family Members’ Perspectives on Family and Social Support Available to Suicidal Patients, and Health Systems’ Interactions and Responses to Suicide Cases in Alberta: Protocol for a Quantitative Research Study
Source: JMIR Res Protoc. 2020 Nov 24;9(11):e19112. doi: 10.2196/19112 (PMC7723743; doi:10.2196/19112)
Supplement: Multimedia Appendix 1 [file resprot_v9i11e19112_app1.docx]

**Family Members’ Perspectives of Family and Social Support available to Suicidal Patients, the Health Systems Interactions and Responses to Suicide Cases in Alberta - Quantitative Research Study Survey**

|  | | | | | | | | | | | | | | | | | | |
| --- | --- | --- | --- | --- | --- | --- | --- | --- | --- | --- | --- | --- | --- | --- | --- | --- | --- | --- |
| **Family Member’s Demographic Information:** | | | | | | | | | | | | | | | | | | |
| **1: What is your age?** | | | | | | | | | | | | | | | | | | |
|  | 18-24 |  | | 25-34 | |  | | 35-44 |  | | | 45-54 | | |  | 55-64 |  | 65 and older |
| **2: What is your gender?** | | | | | | | | | | | | | | | | | | |
|  | Male |  | Female | |  | | Gender diverse | | |  | | | | If gender not listed, please specify: | | | | |
|  | | | | | | | | | | | | | | | | | | |
| **3: How would you describe your ethnicity?** (i.e., the cultural group or family background with which you most closely identify)? | | | | | | | | | | | | | | | | | | |
|  | Indigenous (i.e., First Nations, Aboriginal, Natives, Metis, and Inuit) | | | | | | | | | |  | | African/Caribbean | | | | | |
|  | European/Caucasian | | | | | | | | | |  | | Asian | | | | | |
|  | Prefer not to disclose | | | | | | | | | |  | | Latin American | | | | | |
|  | Do not know | | | | | | | | | |  | | Middle Eastern | | | | | |
|  | If ethnicity is not listed, please specify: | | | | | | | | | |  | |  | | | | | |
|  | | | | | | | | | | | | | | | | | | |

|  | | | | | | | | | | | | | | | | | | | | | | |  |
| --- | --- | --- | --- | --- | --- | --- | --- | --- | --- | --- | --- | --- | --- | --- | --- | --- | --- | --- | --- | --- | --- | --- | --- |
| **4: What is the highest level of education you have completed?** | | | | | | | | | | | | | | | | | | | | | | | |
|  | | 8th grade or less | | | | | | | | | |  | | Some High School | | | | | | | | | |
|  | | High School degree or equivalent | | | | | | | | | |  | | Post-Secondary | | | | | | | | | |
|  | | Post graduate degree  (Master’s degree, Doctorate, other) | | | | | | | | | |  | | Some Post-Secondary | | | | | | | | | |
|  | | Other, please specify | | | | | | | | | |  | |  | | | | | | | | | |
|  | | | | | | | | | | | | | | | | | | | | | | | |
| **5: What is your current employment status?** | | | | | | | | | | | | | | | | | | | | | | | |
|  | | Employed full-time | | | | | | | | | |  | | Unemployed with social assistance income | | | | | | | | | |
|  | | Employed part-time | | | | | | | | | |  | | Unemployed with other income | | | | | | | | | |
|  | | Self-employed | | | | | | | | | |  | | Unemployed with no other income | | | | | | | | | |
|  | | Student | | | | | | | | | |  | | Retired | | | | | | | | | |
|  | | If employment status not listed, please specify: | | | | | | | | | |  | | Unable to work /specify why: | | | | | | | | | |
|  | | | | | | | | | | | |  | | | | | | | | | | | |
| **6: What is your marital status?** | | | | | | | | | | | | | | | | | | | | | | | |
|  | | Single (never married) | | | | | | | | | |  | | Divorced | | | | | | | | | |
|  | | Married/Common law | | | | | | | | | |  | | Separated (not divorced) | | | | | | | | | |
|  | | In a relationship but neither married  nor common law | | | | | | | | | |  | | Widowed | | | | | | | | | |
| **7: What is your relationship to the person who died by suicide?** | | | | | | | | | | | | | | | | | | | | | | | |
|  | | Parent | | | | | | | | | | | | | | | | | | | | | |
|  | | Spouse/Partner | | | | | | | | | | | | | | | | | | | | | |
|  | | Son/Daughter | | | | | | | | | | | | | | | | | | | | | |
|  | | Sibling | | | | | | | | | | | | | | | | | | | | | |
|  | | Uncle/Aunt | | | | | | | | | | | | | | | | | | | | | |
|  | | Grandparent | | | | | | | | | | | | | | | | | | | | | |
|  | | Other, please specify: | | | | | | | | | | | | | | | | | | | | | |
| **Demographic Information of the Patient (The person who died by suicide):** | | | | | | | | | | | | | | | | | | | | | | | |
| **8: Age:** | | | | | | | | | | | | | | | | | | | | | | | |
|  | 18-24 | |  | | 25-34 | |  | | 35-44 |  | | 45-54 | |  | | 55-64 | | |  | 65 and older | | | |
| **9: Gender:** | | | | | | | | | | | | | | | | | | | | | | | |
|  | Male | | |  | Female |  | | Gender-diverse | | | | | | |  | | | | If gender not listed, please specify: | | | | |
| **10: Highest level of education:** | | | | | | | | | | | | | | | | | | | | | | | |
|  | 8th grade or less | | | | | | | | | | | |  | Some High School | | | | | | | | | |
|  | High School degree or equivalent | | | | | | | | | | | |  | Post-Secondary | | | | | | | | | |
|  | Post graduate degree  (Master’s degree, Doctorate, other) | | | | | | | | | | | |  | Some Post-Secondary | | | | | | | | | |
|  | Other, please specify: | | | | | | | | | | | | | | | | | | | | | | |
| **11: Employment status:** | | | | | | | | | | | | | | | | | | | | | | | |
|  | Employed full-time | | | | | | | | | | | |  | Unemployed with social assistance income | | | | | | | | | |
|  | Employed part-time | | | | | | | | | | | |  | Unemployed with other income | | | | | | | | | |
|  | Self-employed | | | | | | | | | | | |  | Unemployed with no other income | | | | | | | | | |
|  | Student | | | | | | | | | | | |  | Retired | | | | | | | | | |
|  | | If employment status not listed, please specify: | | | | | | | | | | |  | Unable to work /specify why: | | | | | | | | | |
|  | | | | | | | | | | | | | | | | | | | | | | | |
| **12: Marital status:** | | | | | | | | | | | | | | | | | | | | | | | |
|  | | Single (never married) | | | | | | | | | | |  | Divorced | | | | | | | | | |
|  | | Married/Common law | | | | | | | | | | |  | Separated (not divorced) | | | | | | | | | |
|  | | In a relationship but neither married  nor common law | | | | | | | | | | |  | Widowed | | | | | | | | | |
| **13: Does the patient consider him/herself within one of the following vulnerable groups with discrimination experience?** | | | | | | | | | | | | | | | | | | | | | | | |
|  | | Gender-diverse – please specify: | | | | | | | | | | | | | | | | | | | | | |
|  | | Ethnicity | | | | | | | | | | | | | | | | | | | | | |
|  | | Religious minority – please specify: | | | | | | | | | | | | | | | | | | | | | |
|  | | Other– please specify: | | | | | | | | | | | | | | | | | | | | | |
|  | | | | | | | | | | | | | | | | | | | | | | | |
| **14: From which health zone did the patient receive health care?** | | | | | | | | | | | | | | | | | | | | | | | |
|  | | **Edmonton zone** (Edmonton, Beaumont, Devon, Leduc, St. Albert, Spruce Grove, Stony Plain, Morinville, Fort Saskatchewan, Sherwood, Thorsby, Kapasiwin--------etc) | | | | | | | | | | | | | | | | | | | | | |
|  | | **Calgary zone** (Calgary, Airdrie, Banff, Black Diamond, Canmore, Cochrane, Cremona, Chestermere, Clarisholm, Strathmore, Gleichen, High River, Vulcan, Nanton, Stavely--------etc) | | | | | | | | | | | | | | | | | | | | | |
|  | | **Central zone** (Red Deer, Breton, Dryton Vally, Ponoka, Camrose, Windfield, Rimbey, Bently, Eckville, Sylvan lake, Sundre, Olds, Two Hills, Three Hills, Drumheller, Hanna, Trochu, Elnora, Innisfail, Lacombe, Blackfalds, Bashaw, castor, galahad, Coronation, Consort, Provost, Killam -etc) | | | | | | | | | | | | | | | | | | | | | |
|  | | **South zone** (Lethbridge, Medicine Hat, Irvine, Redcliff, Brooks, Bassano, Vauxhall, Taber, Bow Island, Fort Macleod, Blairmore, -------etc) | | | | | | | | | | | | | | | | | | | | | |
|  | | **North zone** (Fort McMurry, Grand Prairie, Slave Lake, Athabasca, Lac La Biche-----------etc) | | | | | | | | | | | | | | | | | | | | | |
| **15: Was the patient diagnosed with a mental disorder/illness before completing suicide?** | | | | | | | | | | | | | | | | | | | | | | | |
|  | | No | | | | | | | | | | | | | | | | | | | | | |
|  | | Yes | | | | | | | | | | | | | | | | | | | | | |
|  | | Don’t know | | | | | | | | | | | | | | | | | | | | | |
| **16: If yes to Q 15, please tell us which mental disorder/illness:** | | | | | | | | | | | | | | | | | | | | | | | |
|  | | Depression | | | | | | | | | | |  | Schizophrenia/Psychosis | | | | | | | | | |
|  | | Anxiety | | | | | | | | | | |  | Substance abuse (illicit drugs, recreational substances, prescription medications) | | | | | | | | | |
|  | | Personality disorder | | | | | | | | | | |  | Alcohol abuse | | | | | | | | | |
|  | | Bipolar Disorder | | | | | | | | | | |  | Don’t know | | | | | | | | | |
|  | | Other mental illness, please specify: | | | | | | | | | | | | | | | | | | | | | |
| **17: Did the patient have relationship problems in the months/weeks leading to completing suicide?** | | | | | | | | | | | | | | | | | | | | | | | |
|  | | Yes, relationship problems | | | | | | | | | | | | | | | | | | | | | |
|  | | Yes, loss of a partner/family member | | | | | | | | | | | | | | | | | | | | | |
|  | | No | | | | | | | | | | | | | | | | | | | | | |
|  | | Don’t know | | | | | | | | | | | | | | | | | | | | | |
| **18: As far as you are aware, had the patient attempted suicide in the past before actually dying by suicide?** | | | | | | | | | | | | | | | | | | | | | | | |
|  | | Never | |  | Once |  | | Twice | | |  | | Three times | | | |  | Four times | | |  | Five times or more | |

| **Please mark your response with the following:**  **Did the patient:** | | **Yes** | **No** | **Don’t Know** |
| --- | --- | --- | --- | --- |
| **19** | Receive treatment from local addiction and mental health services? |  |  |  |
| **20** | Have chronic physical health problem(s)? |  |  |  |
| **21** | Have problems with excess alcohol use? |  |  |  |
| **22** | Have problems with prescription medications? |  |  |  |
| **23** | Have problems with illicit drug use? |  |  |  |
| **24** | Have feelings of hopelessness in the weeks and months prior to completing suicide? |  |  |  |
| **25** | Attend or interact with local addiction and mental health providers in the month before completing suicide? |  |  |  |
| **26** | Have a Family Doctor? |  |  |  |
| **27** | Visit their family doctor in the month prior to completing suicide? |  |  |  |
| **28** | Interact with the Emergency Medical Services or call the Mental Health Crisis line in the month before completing suicide? |  |  |  |
| **29** | Attend the Emergency Department in the month before completing suicide? |  |  |  |
| **30** | Become hospitalized on a psychiatric ward in the month before completing suicide? |  |  |  |
| **After the patient died by suicide:** | |  |  |  |
| **31** | Did their family doctor contact their next-of-kin to express condolences and/or offer support to the family? |  |  |  |
| **32** | Did a staff member from the local mental health team contact their next-of-kin to express condolences and/or offer support to the family? |  |  |  |
| **33** | Did any other staff member from the health service (not mental health) contact their next-of-kin to express condolences and/or offer support to the family? |  |  |  |

| **Health system Information** | | | | | | | |
| --- | --- | --- | --- | --- | --- | --- | --- |
|  | **Please mark your level of agreement with the following:** | **Strongly**  **Disagree** | **Disagree** | **Neutral** | **Agree** | **Strongly**  **Agree** | **Don’t Know** |
| **In the months/weeks/days leading up to when the patient died by suicide:** | | | | | | | |
| **34** | The staff at local mental health services who interacted with them were supportive and acted professionally. |  |  |  |  |  |  |
| **35** | The family doctor was supportive and acted professionally. |  |  |  |  |  |  |
| **36** | The staff of the emergency medical services who interacted with the patient were supportive and acted professionally. |  |  |  |  |  |  |
| **37** | The staff of the health services involved family in the patient’s treatment plan. |  |  |  |  |  |  |
| **The patient received support from:** | | | | | | | |
| **38** | The health services to address mental health issues. |  |  |  |  |  |  |
| **39** | The health services to address addiction issues. |  |  |  |  |  |  |
| **40** | Social services to address social issues. |  |  |  |  |  |  |
| **If I should encounter a crisis in the future that made me suicidal, I feel confident I will receive the help I need from:** | | | | | | | |
| **41** | Staff of the emergency medical services/mental health crisis team. |  |  |  |  |  |  |
| **42** | My family doctor. |  |  |  |  |  |  |
| **43** | Community mental health services. |  |  |  |  |  |  |
| **44** | Staff of a psychiatric ward. |  |  |  |  |  |  |
| **45** | I trust the healthcare services in Alberta. |  |  |  |  |  |  |
| **46** | I am aware of the mental health crisis line and other supports available to me if I should experience a crisis that made me suicidal. |  |  |  |  |  |  |
| Is there anything else you would like to share with us about this section? | | | | | | | |

| **Family/Societal Factors** | | | | | | | |
| --- | --- | --- | --- | --- | --- | --- | --- |
| **Please mark your level of agreement with the following:** | | **Strongly**  **Disagree** | **Disagree** | **Neutral** | **Agree** | **Strongly**  **Agree** | **Don’t Know** |
| **The following challenges affected the patient’s mental health and contributed to dying by suicide:** | | | | | | | |
| **47** | Financial challenges |  |  |  |  |  |  |
| **48** | Legal challenges |  |  |  |  |  |  |
| **49** | Relationship problems |  |  |  |  |  |  |
| **50** | Family problems |  |  |  |  |  |  |
| **51** | Work stressors |  |  |  |  |  |  |
| **52** | physical health problems |  |  |  |  |  |  |
| **Other factors and consideration:** | | | | | | | |
| **53** | The patient shared their problems with members of the family prior to dying by suicide. |  |  |  |  |  |  |
| **54** | The patient received help from the family prior to dying by suicide. |  |  |  |  |  |  |
| **55** | Stigma (e.g. a mark of disgrace, shame and dishonor) prevented the patient from seeking help for mental health and other problems. |  |  |  |  |  |  |
| **56** | I seek help from my family when I need it without any fear of negative consequences. |  |  |  |  |  |  |
| **57** | The history of suicide happened in my family makes me have suicidal thoughts. |  |  |  |  |  |  |
| **58** | There are many types of activities in my community which keep me busy and away from suicidal thinking. |  |  |  |  |  |  |
| **Is there anything you would like to share with us about this section?** | | | | | | | |
